# Theoretical Simulation of the Near-Field Probe for Non-Invasive Measurements on Planar Layers with Biological Characteristics

**DOI:** 10.3390/bioengineering7040149

**Published:** 2020-11-19

**Authors:** Aleksandr Gorst, Kseniya Zavyalova, Vladimir Yakubov, Aleksandr Mironchev, Andrey Zapasnoy

**Affiliations:** Radiophysics Faculty, Tomsk State University, Tomsk 634050, Russia; aleksandr.gorst@mail.tsu.ru (A.G.); yvlp@mail.tsu.ru (V.Y.); mironchev42@mail.ru (A.M.); zapas@mail.tsu.ru (A.Z.)

**Keywords:** near-field sensor, non-invasive, microwave sensor, biological tissues, modeling, simulation

## Abstract

The article presents the design of the near-field probe, which is a combined emitter (a combination of a symmetric dipole and an annular frame). The design of the probe allows forming a prolonged zone of the near-field. This effect can be used for in-depth penetration of the field in media with high absorption, without loss of information. Particular attention in this article is given to a detailed study of the interaction of the field created by this probe on plane-layered biological media. A theoretical analysis of the interaction of the electromagnetic field was carried out in a wide frequency band with a model plane-layer biological medium containing blood vessels of shallow depth using the proposed probe design. Conclusions are drawn about the depth of penetration of a useful signal into different media-analogs of biological tissue. This study is necessary to consider the possibility of using this probe for non-invasive measurements of blood glucose concentration. The studies were carried out using numerical simulation in the CST (Computer Simulation Technology) Microwave Studio environment. All biological tissues were simulated over a wide frequency range from 10 MHz to 10 GHz.

## 1. Introduction

Diabetes mellitus is a significant public health problem. According to the World Health Organization and the International Federation of Diabetes, the prevalence of this disease is third after cardiovascular and oncological diseases and over the past few decades, the number of cases and the prevalence of diabetes have been steadily increasing [1,2,3]. Moreover, this disease is still considered incurable. Diabetes mellitus and its complications are one of the leading causes of disability and mortality in the population, including those of working age.

Great importance in the fight against diabetes is given to the prevention of the disease. Frequent and accurate self-monitoring of blood glucose levels (glycemia) is important for the timely correction of all deviations in the level of glycemia [4].

Self-monitoring of blood glucose levels is currently carried out, as a rule, using the usual method of finger puncture (invasive methods), and is the most common method for determining glucose levels today [5]. However, this method has several significant disadvantages (pain, inconvenience, especially for patients who need to check the blood glucose level several times a day, the risk of infection), which limit the widespread use of this method, and result in a lack of proper frequency glucose level measurement and, consequently, makes it difficult to compensate for diabetes [6]. These factors make non-invasive methods of glucose monitoring very popular.

Currently, numerous studies are aimed at developing an effective non-invasive technology for analyzing blood glucose concentration [7]. This opportunity will allow the prevention and early diagnosis of the population to identify disorders of carbohydrate metabolism, which will lead to a decrease in the number of diabetes mellitus diseases and will also reduce expenses on consumables. To date, a large number of different approaches have been proposed for non-invasive determination of glucose levels, ranging from optical analysis methods [8] to ultrasonic and bio-impedance methods, and some scientists are developing various biosensors [9]. However, despite the intensive development of non-invasive methods for determining glucose levels [10], a ready-made technology has not yet been developed, the accuracy of the created non-invasive glucometers leaves much to be desired, and their application in clinical practice is extremely difficult.

The complexity of the task is explained by a lot of factors affecting the recorded signal, which must be taken into account and optimized to obtain an effective device. These factors range from the effect of the skin to the percentage of glucose [11]. In fact, the question boils down to extracting useful information from noise, or to identifying an empirical relationship between the observed phenomenon and glucose level. The optimization of these factors has been studied over the past 20 years; however, unambiguous and final results have not yet been achieved, which does not allow these technologies to fully “occupy” their market [12,13].

In this paper, we propose studying the features of the near-field interaction of electromagnetic radiation in a wide frequency band (near-field radio wave diagnostics or microscopy) with the diagnosed plane-layered biological medium to further explore the possibility of creating, on this basis, a new effective non-invasive glucometer technology.

When applying electromagnetic radiation to a biological medium, some of the energy of electromagnetic radiation is reflected back from the surface of the tissue. The remaining energy of electromagnetic radiation passes through the surface to the underlying layers. At the same time, part of this transmitted energy is absorbed by some tissues, part is reflected back and recorded by the electromagnetic wave probe. The absorption, reflection, and passage of electromagnetic waves through the tissue (biological environment) depend on the dielectric characteristics, which in turn depend on the concentration of glucose.

A review of existing solutions for electromagnetic sensors and antennas (microstrip patch antennas, spiral microstrip resonator, open-loop microstrip resonator, split-ring resonators, patch resonators, dielectric resonator antennas, etc.) shows a rather low accuracy of the proposed solutions for measuring glucose concentration [14,15,16,17,18,19]. 

Therefore, Jang, C., Park, J.K., Lee, H.J., Yun, G.H., Yook, J.G. [15] considered a resonant sensor based on a complimentary split-ring resonator approach based on the consideration of the transmitted signal; the setup itself was a sensor with the liquid under study located directly above the main radiation. The glucose level was from 0 to 22 mmol/L, with a step of 5.5 mmol/L. The results obtained showed an extremely weak sensor response to glucose changes, namely, a difference of 0.004 dB.

A similar sensor was also considered in the article [14]. As in the previous case, the resonant method was used, but in this case, both the transmitted signal and the reflected one were considered. The range of glucose levels ranged from 2.7 to 11.1 mmol/L, in 2.7 mmol/L steps. The difference in signals upon changing the glucose level averaged 0.015 dB. Taking into account the absence of other highly absorbing media (skin, muscles), this result is extremely small for determining the glucose level in reality.

A detailed experiment taking into account all biological tissues was carried out and shown in the article [19]. The sensor was fixed on the wrist, the temperature of the room in which the ten-hour experiment took place was controlled and amounted to 23.8 °C. The sensor itself was an RLC—a resonant frequency circuit. The data obtained from the sensor differed greatly from the real values of glucose with a sharp change in blood sugar. With a small change, the obtained values were similar to the real ones.

We consider that this is primarily due to the presence of a protective skin and muscle cover of a human. Overcoming this cover is a kind of “stumbling block” on the way to creating an effective non-invasive device for assessing blood glucose levels. As a rule, it is the skin and the parameters of the internal environment that make significant errors in the measured data.

In this paper, the authors propose to investigate the possibility of using a near-field electromagnetic probe for research biological media. Unlike other studies in this work, special attention is paid to studying the influence of various layers of tissue on the measurements of the proposed probe. We believe that these studies are of great interest and can be used to create an effective technology for non-invasive measurement of glucose concentration.

## 2. Materials and Methods 

### 2.1. Selection of a Biological Medium

Before creating a plane-layered medium, it is necessary to consider the most optimal part of the body from the point of view of applying a probe for a return signal with minimal noise. Modern research on creating tools for measuring blood glucose concentration is mainly aimed at creating a sensor located on the wrist and forearm [20,21,22]. This approach is associated with a large number of lymphatic vessels located at a shallow depth of bedding, as well as a thin fat layer, in comparison with other parts of the body. Therefore, in the article, it is proposed to consider as biological media: Skin—as the first organ through which the signal should pass, then—blood, fat, muscles, and bone.

The skin is divided into epidermis and dermis (the main component of this organ), as well as the hypodermis, which forms the cell space, which includes fatty deposits and blood vessels. The thickness of the epidermis (first layer of the skin) of most parts of the body is 0.07–0.12 mm, and on the palmar surfaces of the hands and plantar surfaces of the feet reaches 0.8–1.4 mm. The water content in the surface layer is only 10% by weight of the cells. The area of sweat and sebaceous glands, hair follicles in different parts of the body is not the same: 0.5% of the skin surface [23].

Dermis is the middle and main layer of the skin. It is located between the epidermis and hypodermis. The thickness of the dermis is from 0.5 to 5 mm. Dermis maintains a constant temperature of the body—about 37 °C. The dermis has a large number of blood and lymph vessels, sensitive nerve endings. There are sebaceous and sweat glands and also hair roots. There are smooth muscle cells, sometimes forming small bundles. The water content in the layer reaches 70% by weight of the cells.

The thickness of fat tissue (the lower layer of the skin) varies from a few millimeters to several centimeters. On the palms, stomach, and buttocks, it is well developed and has a large thickness in relation to such parts of the body as arms and legs. It contains fat as well as blood vessels [24].

The next biological material is muscle. Muscles consist of a large number of muscle tissue covered with fascia (connective tissue membrane covering organs, blood vessels, nerves and forming cases for muscles in vertebrates and humans; performs supporting and trophic functions). Muscles occupy most of the space of a human limb.

The last material to build a hand is bone. Bone is a solid organ. The bone performs supporting-mechanical and protective functions. The bone structure is porous, which ensures the high permeability of electromagnetic waves.

Table 1 with all the materials considered and their thickness range.

The nature of the interaction of the electromagnetic field with various tissues is determined by their electrical and magnetic properties. For the task of considering the interaction of the near-fields with biological tissue, we analyzed the change in the dielectric constant for each of the above media. The composition of various tissues includes ions, spatially oriented, polar and nonpolar macromolecules of various linear sizes, as well as water dipoles. Different biological tissue contains them in different proportions, so each of them has different dielectric properties and electrical conductivity.

Considering the values of the dielectric constant, it can be concluded which of the materials will introduce the most noise when receiving data. Fat will make a minimum of changes, which increases the chance of obtaining reliable data for different sizes of people. Muscles, like skin, will make the maximum changes in the data obtained. In the case of muscles, this data can be ignored, since the blood vessels and veins are located above these tissues. The skin, on the contrary, is located on top of the vessels and veins; therefore, a very important component is the place for measuring glucose levels. Thus, the probe should be located in the area with the smallest skin thickness and the largest number of vessels and veins.

### 2.2. Near-Field Probe 

Previously, the authors proposed a new approach to the probing of strongly absorbing media [25]. The emitter (antenna) has two spatially distributed zones near it—near and far. Based on previous studies, the border of the near and far zones is quite localized. This boundary is called causal. In the near-field, attenuation (power-law) of the electromagnetic field is not related to the absorption of radiation, but the far-field absorption (exponential) actually occurs. The field inside the causal region is quasistatic, and its phase velocity can significantly exceed the speed of a traveling wave in a given medium, that is, the speed of light in a medium.

This means that the created electromagnetic field becomes a traveling wave only in the far zone (outside the causal region). Due to the fact that the near field attenuates more slowly than the probe radiation in highly absorbing media, the received reflected signal will be much larger in amplitude in comparison with the far-field radiation. A traveling wave propagates with the speed of light in this medium, and in the presence of absorption in the medium, the amplitude of this wave acquires exponential attenuation. In contrast, the attenuation of radiation in the near-field is not exponential even for a medium with absorption but is determined by the power law of decreasing field amplitude with distance. The near-field of the emitter penetrates deep enough. This allows maximizing the signal to noise ratio. This effect is of great interest to create an effective non-invasive glucometry technology. However, it requires an in-depth study of the physical processes that occur during the interaction of electromagnetic fields in the near-field and biological tissues. The size of the near zone depends on the frequency and refractive index of the medium. More details on this theory can be found in the article [25].

**Figure 1:**
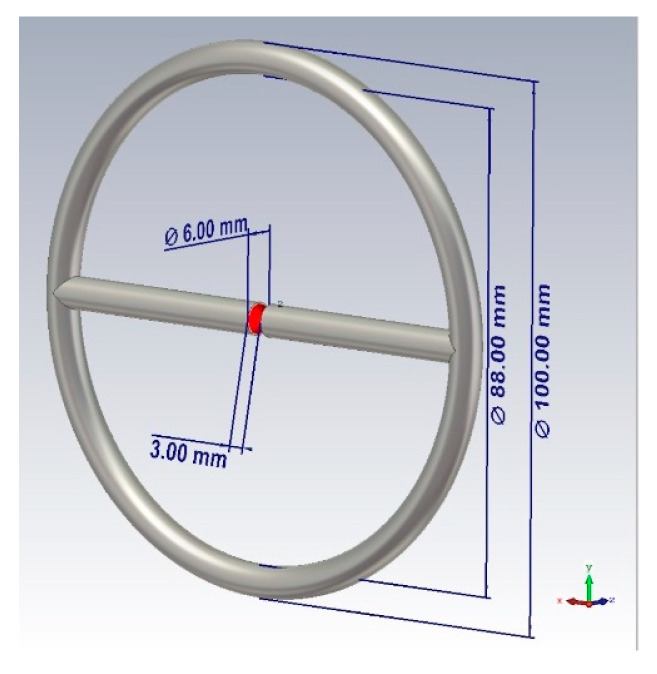
Design of the near-field probe.

Based on the presented theory, the design of the probe shown in Figure 1 was proposed. The sensor is a combined emitter (a combination of a symmetric dipole and an annular frame). The emitter is powered at the input of a symmetric dipole. The outer diameter of the probe is 100 mm, the diameter of the tube is 6 mm, the distance between the left and right arms of the probe is 3 mm.

Directions of the radiation of the electric and magnetic fields coincide, thus the bound energy of the near zone is concentrated in one direction. This fact provides the maximum possible interaction of electromagnetic radiation with the medium when we are probing the objects of study, as a result, we have the best response in the spectral characteristic.

### 2.3. Dielectric Properties of Tissues

**Figure 2:**
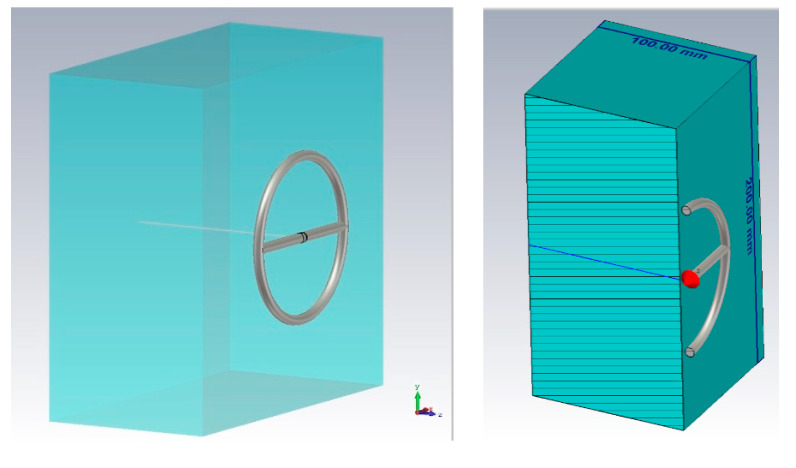
The scheme of the simulation.

The dielectric properties of tissues were investigated. Each type of biological tissue in the model was represented as a layer of 100 mm thick, 200 mm wide, and 200 mm long (Figure 2). This thickness is optimal for studying the penetration of the near-fields into the medium under consideration.

**Figure 3:**
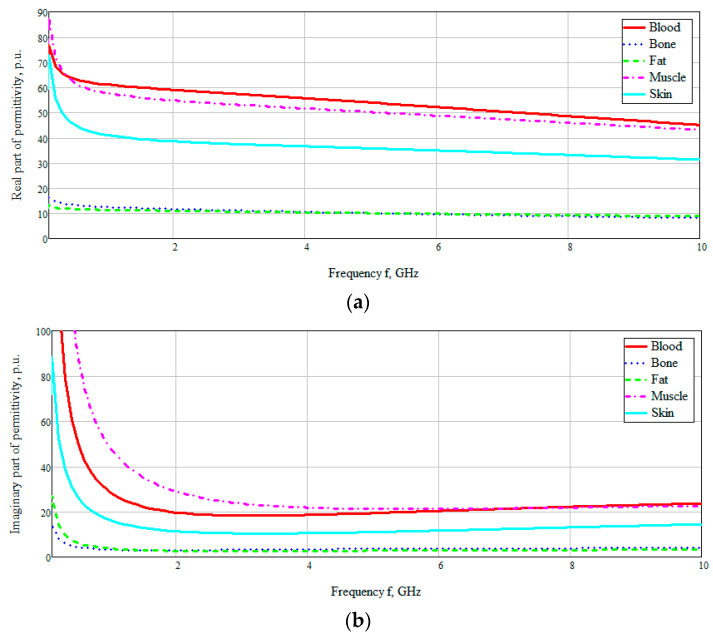
Frequency dependence of the real (**a**) and imaginary (**b**) parts of permittivity of biological media.

In [26] the dielectric properties of tissues (including blood) were experimentally characterized in a wide frequency range from 10 Hz to 20 GHz (Figure 3). The dielectric properties of blood in Figure 3 are based on the Cole–Cole model. The Cole–Cole relaxation model is widely used by researchers to extrapolate the measured permittivity of biological tissues at high frequencies.

We took these values of the dielectric constant of the required biological media, namely, blood, fat, muscles, and bone and imported to CST Microwave Studio. We performed modeling in a wide frequency band (10 MHz–10 GHz) for the subsequent determination of the maximum near-field penetration. When modeling, an open boundary was used with the addition of some additional space between the structure and the applied boundary condition (Open (add space)). The sensor was powered using a discrete port, which is an impedance element (50 Ohms), which also absorbs power and allows you to calculate the S-parameter.

Figure 3 shows the obtained frequency dependences of the values of the real and imaginary parts of the dielectric constants of the taken biological media in the entire investigated frequency range. The data are based on the studies by A. Peyman [27] and C Gabriel [28]. The database used was taken from [29]. According to the presented diagram of the distribution of permittivity, it can be seen that media containing an aqueous solution, such as blood, muscles, skin, have a higher permittivity. Based on this, it can be assumed that electromagnetic waves will attenuate faster in these tissues (blood, muscles, and skin).

**Figure 4:**
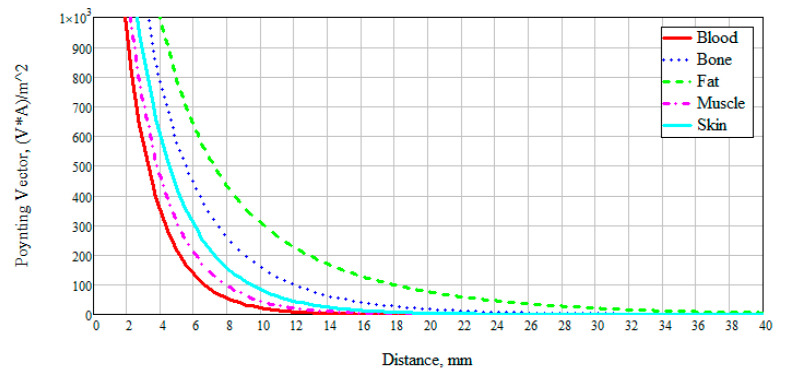
Dependence of the power flux (Poynting vector) on the distance at a frequency of 6 GHz.

We also examined the interaction of electromagnetic near-field with a biological medium based on a power flux (Pointing vector). This characteristic allows seeing how far the field penetrates the test study sample, and also at what distance it attenuates. Figure 4 presents the obtained data on the results of numerical modeling of the power flow for different biological media at 6 GHz. The frequency was chosen based on the thickness of the modeled layer (Figure 2). Since the layer thickness was chosen to be 100 mm, thus, there are two waves (6 GHz corresponds to a wavelength of 50 mm). We can see that the attenuation of electromagnetic waves for blood, muscles, and skin has a similar exponential character. This result is explained by the dielectric properties of these media and the presence of an aqueous solution in them: The muscles are 86.3% water, the blood consists of plasma and shaped elements, where, in turn, about 85% of the plasma is water. The skin also includes sebaceous glands which are two-thirds of water [26].

The power flow of electromagnetic waves attenuates for blood, muscles, and skin at a distance of 30 mm, for bone at 60 mm, and for fat goes beyond 100 mm. This result is positive since when considering the anatomy of the structure of the forearm of the human body, we can observe changes in skin thickness from 0.1 to 1.5 mm, muscles up to 20–25 mm, and blood vessels, as a rule, reach a diameter of no more than 15 mm.

## 3. Analysis of the Interaction of the Electromagnetic Field in a Wide Frequency Band with a Model Plane-Layered Biological Medium 

To conduct this study we focused on the inside of the forearm. There are a large number of lymphatic and arterial vessels, the thickness of which reaches up to 3 mm. These vessels are under the skin and fat layers. The dermis is a part of the skin that consists of connective tissue with some elastic fibers and smooth muscle cells. The thickness of the dermis is not the same for different people. The thickness of the dermis on the forearm varies in the range of 1.0–1.5 mm, and in some places reaches 2.5 mm. The thickness of the subcutaneous fat layer on the lower leg is highly correlated with the thickness of the fat on the thigh, forearm, and shoulder and ranges from 0.6 to 0.7 mm, where 0.1 mm refers to fat tissue located in the skin [22]. The muscle layer of the forearm is an average of 30 mm in diameter. Considering the thickness of the muscle layer in a longitudinal section, we obtain a distance from the muscles to the bone of 15 mm.

**Figure 5:**
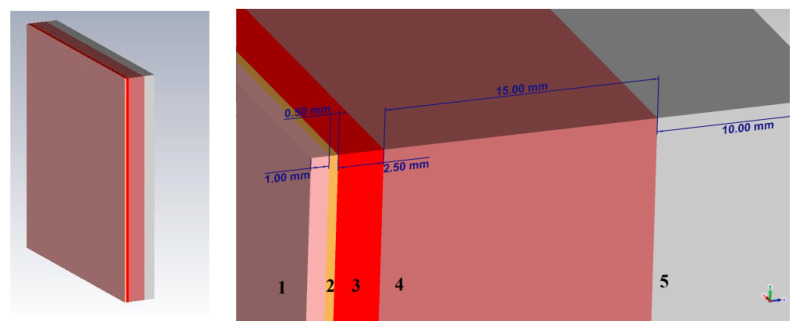
Model of a plane-layered medium with biological tissues (1—skin, 2—fat, 3—blood, 4—muscles, 5—bone).

Thus, when modeling a plane-layered medium close to the structure of the human forearm, we take a skin thickness of 1 mm, a fat layer of 0.5 mm, muscle fibers of 15 mm, and bone tissue of 10 mm thickness. The simulation was performed using the CST Microwave Studio software. A complete environment that mimics the human forearm consists of five main layers: skin, fat, blood, muscles, bone. In CST Microwave Studio, the corresponding thicknesses of each of the biological layers were selected and a model of a plane-layered medium was built. The model is shown in Figure 5.

For a detailed consideration of the impact of the near-field on a planar environment, we looked at various combinations of these media options: (1) Skin, fat, blood; (2) skin, fat, blood, muscles; (3) skin, fat, blood, muscles, bone. Such combinations of biological media allow to more accurately determine which of the biological tissue have the greatest impact on the near-field sensor. The probe described in the upper will be used as a near-field sensor.

**Figure 6:**
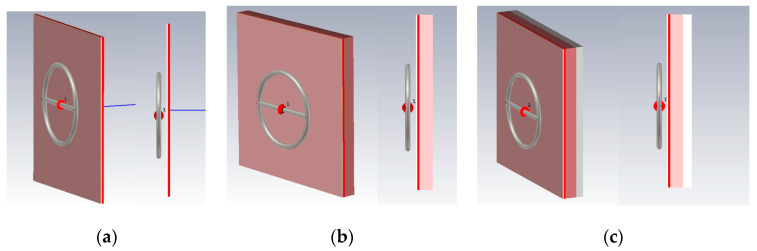
Modeling Combinations 1 (**a**) skin, fat and blood, 2 (**b**) skin, fat, blood, muscles, and 3 (**c**) skin, fat, blood, muscles, bone.

Let us consider Combination 1: Skin, fat and blood. This combination provides the minimum thickness above biological media. Figure 6 shows this model. You can see the near-field probe located at a distance of 6 mm from the test medium (combination 1). The width and length of the plane-layered medium were chosen at 200 mm to completely cover the radiation of the probe. The simulation was carried out in the frequency range 0.01–10 GHz. This range is optimal for the probe when considering the near-field and reflected signal. The following data were measured: The amplitude of the electric and magnetic components of the field and power flux along the line marked in the figure.

**Figure 7:**
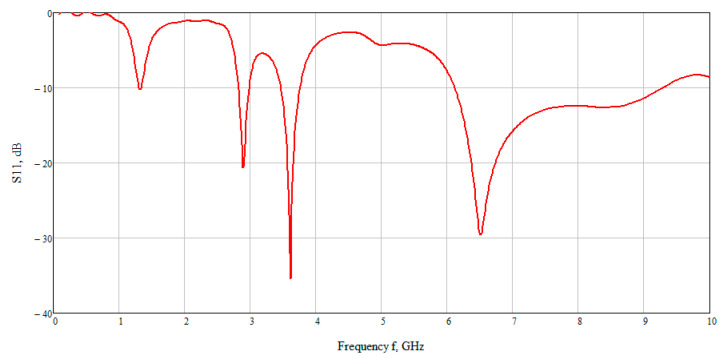
The dependence of the reflected signal (S11) on frequency.

The calculation results are shown in Figure 7 which presented the graph of the reflected signal. This graph allows observing that the best result is obtained at low frequencies. On closer examination, the maximum reflected signal is located in the range 6–7 GHz. Therefore, further we examined the electric and magnetic component of the field at an average frequency of 6.5 GHz.

**Figure 8:**
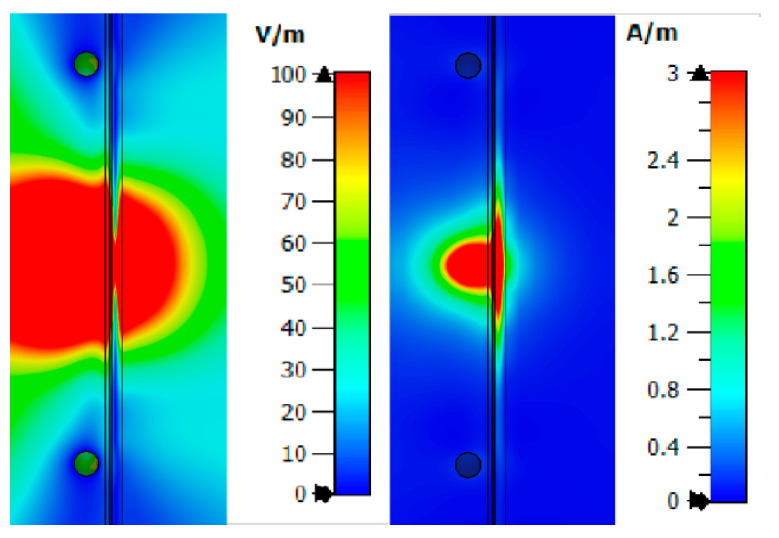
Electric and magnetic fields at a frequency of 6.5 GHz for Combination 1.

From Figure 8, one can observe the past field, the maximum value of which for the studied combination is:

1. Electric field strength E = 110.6 V/m;
2. Magnetic field strength H = 1.63 A/m.

The maximum loss of power occurs in the area of interaction of the near-field with the blood medium. As previously shown, this is associated with large dielectric losses in this medium. Additionally, when considering the field E and H, one can observe zero values, which are shown in the Figure 8.

Thus, we simulated the propagation of the near-field in the upper layers of the human forearm. The analysis showed that the strength of the electric and magnetic components of the field has zero values in the biological medium of the blood. This reduces traversed energy.

**Figure 9:**
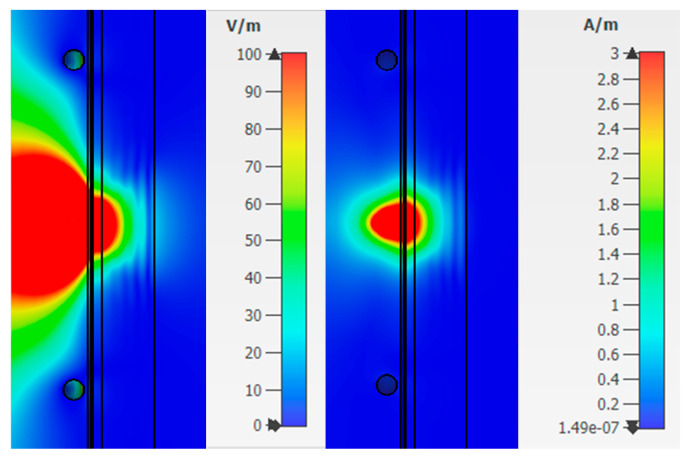
Electric and magnetic fields at a frequency of 6.5 GHz for Combination 2.

Next, Combination 2 was simulated: Skin, fat, blood, and muscle (Figure 6b). Muscles are also affected by diabetes, but less. With the breakdown of sugar (glycogen) not processed in the blood, part of it enters the muscle fibers. As in the previous case, we consider the strength of the electric and magnetic fields (Figure 9) at a frequency of 6.5 GHz. The figures show that the electric and magnetic fields also have zero field marks, due to which we get a lower response of the system.

The value of the field strength behind the biological plane-layered medium is much less than in the case with Combination 1 (skin, fat, blood) and takes values:

1. Electric field strength E = 20.6 V/m;
2. Magnetic field strength H = 0.11 A/m.

This result is explained by the presence of a strongly absorbing medium, thus the near field in such a medium decays more slowly than in open space.

**Figure 10:**
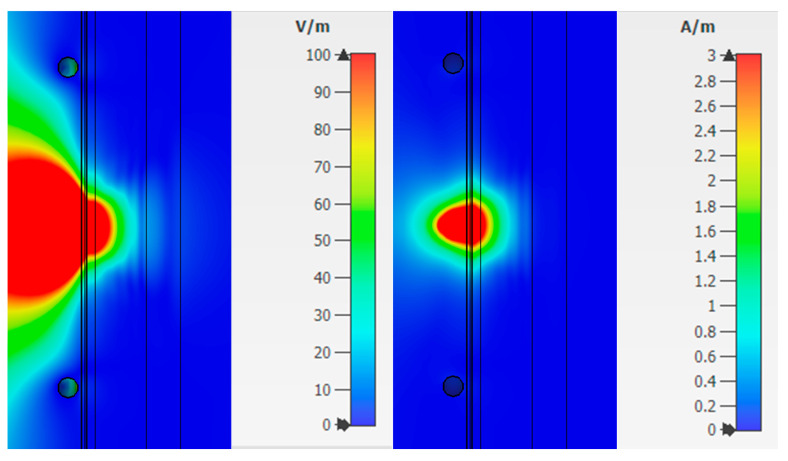
Electric and magnetic field at a frequency of 6.5 GHz for Combination 3.

Combination 3 consists of layers of skin, fat, blood, muscle, and bone (Figure 6c). Bone is not affected by diabetes mellitus, but has a higher dielectric constant than fat, so it is also an important part when considering a flat-layered structure. Figure 10 shows the strength of the electric and magnetic fields for Combination 3.

Analysis of fields for Combination 3 shows that the maximum attenuation and amplitude zeros are concentrated in the muscle area. This effect indicates a weak response of the system in this area. The reflected waves will have little effect on the overall picture taking into account the weak effect of glucose on muscle fibers.

The value of the field strengths behind the biological flat-layered medium with combination 3 takes on the values:

1. Electric field strength E = 7.6 V/m;
2. Magnetic field strength H = 0.02 A/m.

**Figure 11:**
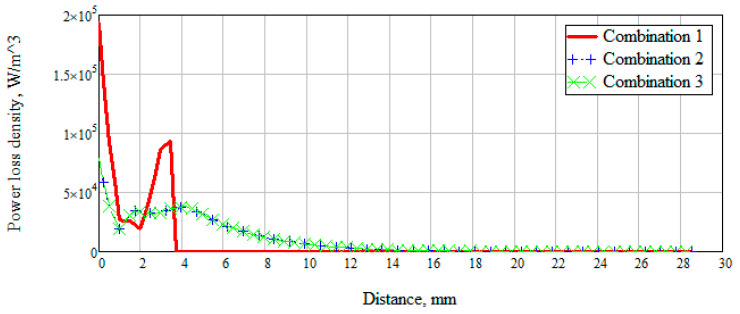
Dependence of power loss density on distance.

There was also a measurement of the power loss density to consider the values of the dissipated electrical and magnetic energy inside biological materials (Figure 11).

Figure 11 shows that when radiation penetrates into the layers of the skin-fat, a large loss of energy happens, which decreases with distance. Wherein losses in the layer with blood increase significantly (Combination 1). When a layer with muscles (Combination 2) is added to the modeling, a sharp change in power loss occurs. It is due to a uniform attenuation of energy throughout the entire layer. Since most of the energy is attenuated in the muscle layer, then already at a distance of 20 mm the losses have a minimum value. Combination 3 is identical to combination 2, which means that the added layer with bone parameters does not affect energy decay.

**Figure 12:**
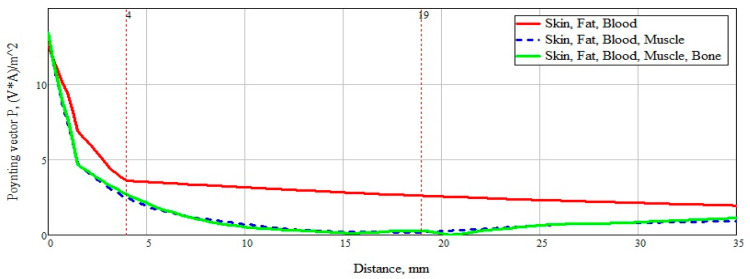
Power flux at a frequency of 6.5 GHz along a straight line for all considered combinations.

**Figure 13:**
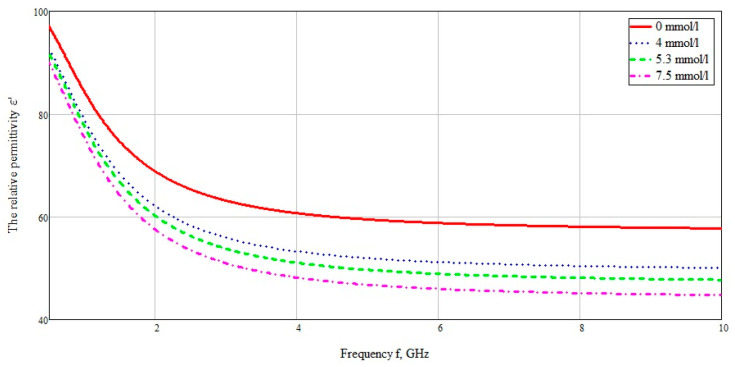
The relative permittivity of blood with different concentration of glucose.

To summarize, it is necessary to more clearly and accurately consider the power flow passing through a plane-layered medium (Figure 12). An analysis of Figure 13 shows the significant effect of a layer consisting of skin, fat, and blood (Combination 1). For clarity, this layer is marked with a marker at a distance of 4 mm from the beginning. The subsequent addition of a muscle layer affects the overall picture of the power flow. This layer is also marked with markers located at a distance of 4 mm and 19 mm. When adding a layer with bone tissue, changes in the power flow are minimal and do not make changes in the overall picture.

The influence on the reflected signal with different concentrations of glucose in the blood layer was also considered. As a sample of biological tissue, the material selected was blood, with various glucose concentrations. To calculate the dielectric constant, we use the data obtained by Benjamin Frier from the University of Ferley Dickinson [26]. Using the modified Cole–Cole equation below:

ε(ω)=Re[ε∞+∑m=141∆εm1+(jωτm)(1−αm)]·[(−0.001445)g+1.145882]+Im[ε∞+∑m=141∆εm1+(jωτm)(1−αm)+σijωε0]

where, $\varepsilon_{\infty }$—“infinite frequency” of the dielectric constant, ω—angular frequency, τ—time constant, α—exponent parameter takes values from 0 to 1, ε(ω)—complex dielectric constant.

The data were used for concentrations of 72, 95, and 134 mg/dL, when converted to the European measurement standard 4, 5.3, and 7.5 mmol/L, respectively. The data used are selected from the calculation of the minimum and maximum glucose concentration for a person who does not suffer from excess blood sugar, as well as the glucose concentration after a meal. The dielectric data was imported into CST Microwave studio software. The plotted graph in the frequency range from 10 MHz to 10 GHz is shown in Figure 13.

**Figure 14:**
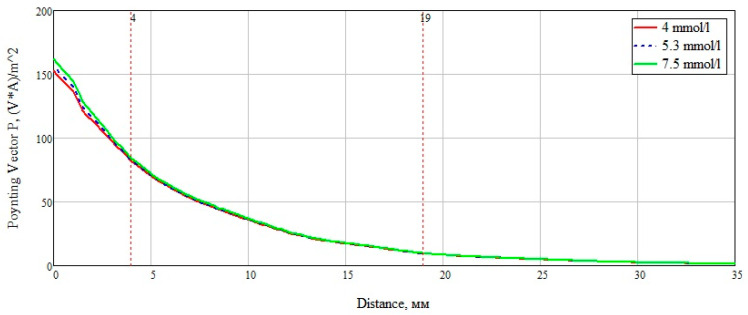
Power flow at 6.5 GHz along a straight line.

After analyzing the Poynting vector along the straight line passing through the center of the structure, from Figure 14, it can be concluded that the maximum difference in the power flow occurs in the layer of skin, fat, blood, up to the marker at the 4 mm point. This technique for measuring glucose concentration is optimal for shallow vessels.

**Figure 15:**
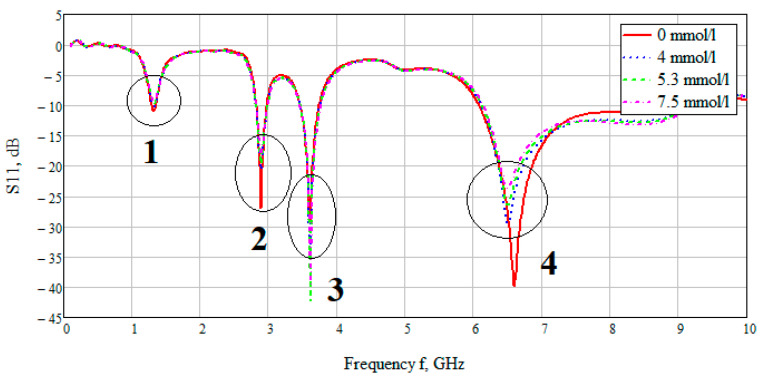
Parameter S11 for the biological environment blood with different glucose concentration. Resonant values of S11 are marked with areas 1,2,3,4 at frequencies of 1.3, 2.9, 3.6, 6.5 GHz, respectively.

The S-parameter of three cases (4, 5.3, 7.5 mmol/L) was considered to compare the effect of glucose concentration. Figure 15 shows the value of the reflected signal at various glucose concentrations, there are four resonant regions.

**Figure 16:**
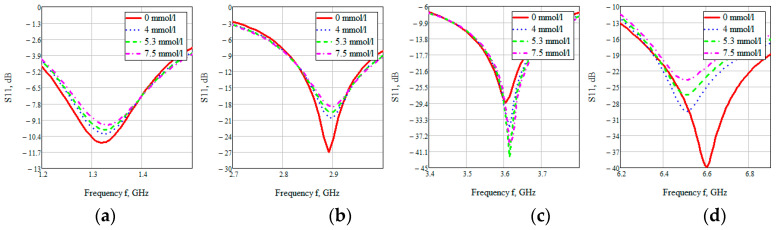
Parameter S11 for the biological environment blood with different glucose concentrations for areas 1 (**a**), 2 (**b**), 3 (**c**), 4 (**d**).

Looking at each of the areas separately (Figure 16), you can see that three of them are not confidence areas. Thus, in the region one the values of the reflected signal for each of the concentrations are hardly distinguishable, namely at 0 mmol/L-0.9 dB, 4 mmol/L-10.2 dB, 5.3 mmol/L-9.9 dB, and 7.5 mmol/L-9.5 dB. With such a weak difference between the reflected signals, it is impossible to distinguish between glucose values.

The second area is also poorly distinguishable in signal level at different glucose concentrations, namely, 0 mmol/L-27 dB, 4 mmol/L-20.6 dB, 5.3 mmol/L-19.5 dB, 7.5 mmol/L-18.5 dB. On average, the difference between concentrations was 1 dB.

In the third area, the signals are easily distinguishable, but the values of the reflected signal for each of the concentrations are lined up in the wrong order, namely, 0 mmol/L→4 mmol/L→7.5 mmol/L→5.3 mmol/L.

The far right range is not more suitable for further investigation. In region four, there is a large difference between the reflected signals, namely 0 mmol/L-40 dB, 4 mmol/L-29.5 dB, 5.3 mmol/L-26.4 dB, 7.5 mmol/L-23.7 dB. Thus, the difference between the reflected signal for different glucose concentrations averaged 3 dB.

**Figure 17:**
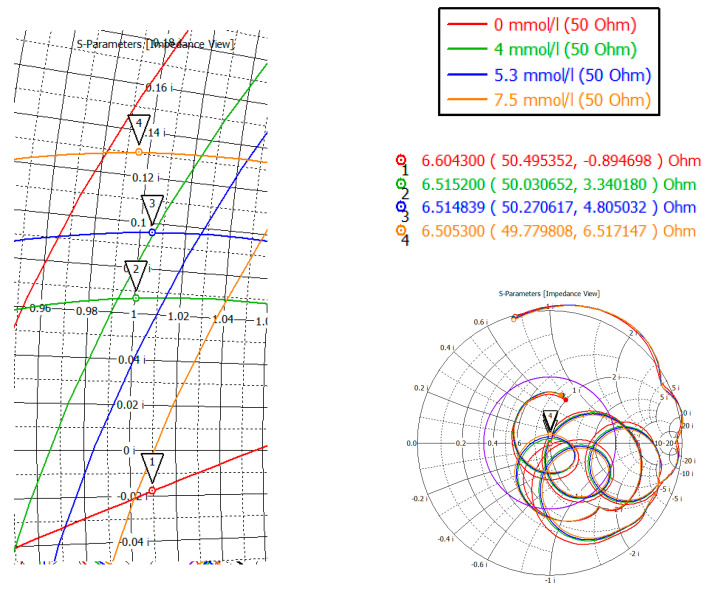
Complex resistance for the biological environment of blood with different concentration of glucose for region four.

To investigate the complex resistance in area four, the Smith chart was considered (Figure 17). Markers mark the minima of the region four. As can be seen from the indicators, the value of the active part is 50 ± 0.5 Ohm, the reactive part is also small in the region of 6 Ohm.

In this way, the maximum effect on the power flows and; therefore, on the reflected signal, has a layer consisting of skin, fat, and blood. This result is explained by a large absorption of the skin and blood. Muscle tissue also affects the reflected signal, only in a less significant form (the graph takes on a character with exponential attenuation). A bone tissue layer does not affect the flow of power passing through the structure.

## 4. Conclusions 

Modeling and simulation of the proposed design of the near-field sensor show the possibility of using this design for non-invasive measurement, in particular for measuring glucose concentration in biological tissues. We focused on a detailed study of the effect of the electromagnetic field generated by this near-field sensor on biological tissues. This study is essential for the best understanding of the tissue interaction of this sensor. In the future, this research will be useful for understanding the physical picture and creating real sensors for biological tissues.

As a result of the work done, it was possible to determine that the maximum effect on the power flow, and therefore on the reflected signal, has a layer consisting of skin, fat, and blood. This result is explained by a large absorption of the skin and blood. At the same time, the obtained data on the depth of penetration of the power flow into the biological fat medium indicates the possibility of taking measurements for people with various degrees of obesity, since it has a weak effect on the received signal, regardless of thickness. Biological muscle tissue has a negligible effect on the reflected signal. A study of bone tissue showed that wave attenuation occurs at an even slower rate than in muscles.

Thus, considering the human forearm, which in the general case is a sequence of layers of skin, fat, blood vessels, muscles, and bone, it can be concluded that, to take into account the concentration of glucose in the blood, it will suffice to consider and pay special attention to the reflection of the useful signal from the first three layers (skin, fat, blood vessels). The obtained depth of signal penetration into these media suggests that the proposed sensor design will allow sensing the concentration of glucose in the blood with high accuracy.

The results are new and have not been previously investigated by anyone. This study is interesting and is of great importance for further investigation of this sensor design as a possible effective tool for the analysis of soft biological tissues in general. The effect of glucose on both the power flow and the reflected signal was also investigated. Thus, the use of a near-field probe makes it possible to measure glucose levels with high accuracy, provided that the vessels are not deeply buried. 

Further development will be aimed at confirming the obtained modeling data on real experiments. We plan to minimize the device for more convenient operation. It will also allow it to be tightly fixed on the human body. We also will investigate separately the effect of temperature, hydration, and other factors on the received signal in real experiments (with a created model or prototype). 

## Figures and Tables

**Table 1:** Thickness of materials.

Biological Tissue	Thickness, mm
Fat	0.1–150
Muscles	5–100
Skin	0.57–6.4
Bone	12–90
